# Knowledge and Awareness of Oral and Perioral Piercing and Its Complications Among the Population in Riyadh

**DOI:** 10.7759/cureus.41930

**Published:** 2023-07-15

**Authors:** Hajer Aldulaijan, Bader Fatani, Nawaf Alfhaed, Mohammed Alquhayz, Abeer Alnafea, Reem Alhokair, Arwa Talakey

**Affiliations:** 1 Periodontics and Community Dentistry, College of Dentistry, King Saud University, Riyadh, SAU; 2 College of Dentistry, King Saud University, Riyadh, SAU; 3 College of Medicine, King Saud University, Riyadh, SAU; 4 General Dentistry, Ministry of Health, Riyadh, SAU; 5 General Dentistry, Private Health Care, Riyadh, SAU

**Keywords:** oral hygiene, complications, oral health, perioral piercing, oral piercing

## Abstract

Background and objective

Body piercing was a cultural custom associated with religious or ceremonial rites in antiquity. Currently, it has grown in popularity among teenagers and young people as a form of self-expression. Young adults are now frequently seen with body and oral piercings, which can cause several complications. Patients with intraoral piercing often present with poor dental and periodontal health, as well as various complications and side effects. The general public is often poorly informed about the risks they are exposed to after intraoral piercing and the strategies for minimizing them, and even healthcare professionals often have limited knowledge of the risks and complications that may arise after this procedure. To the best of our knowledge, no published data assessing the knowledge of oral and perioral piercing among the population living in Riyadh City, Saudi Arabia are currently available. In light of this, we conducted this study to assess the level of awareness and knowledge of oral piercing and its complications among the population living in Riyadh city and evaluate the correlation of nationality, sex, age, and socioeconomic status (educational level, area of residence, and income) with the level of individual knowledge.

Methods

This was a cross‐sectional observational study with a sample size of 1,099. A confidence interval (CI) of 95%, a standard deviation of 0.5, and a margin of error of 5% were employed to determine the sample size. A structured questionnaire was used as the study tool and was distributed in several residential areas in Riyadh, such as North, South, Middle, Eastern, and Western Riyadh City. The data collection was performed using simple random sampling via electronic questionnaires distributed to participants living in Riyadh.

Results

A total of 1,054 individuals completed the survey. Of these, 95.6% were Saudi nationals. Approximately 85.5% of the participants (n = 901) were women, 52.4% were aged 20-29 years (n = 552), and most participants (62.9%, n = 663) had a low monthly income (<5,000 Saudi Riyals); in terms of residence, the highest number of participants were from the northern region of Riyadh (37.1%, n = 391). Most participants had heard of or seen an oral or perioral piercing (89.1%, n = 939). However, very few of them had received an oral piercing themselves (10.7%, n = 113) or had a family member with an oral piercing (18.7%, n = 197). Participants reported that the most commonly observed site for oral piercing was the lip (29.8%, n = 314), and teenagers were the most common age group with oral piercings (76.3%, n = 804). Regarding the adverse effects, most participants reported being aware of the negative consequences of oral piercing in the mouth (72.2%, n = 761). Sex and age were the only factors that showed a significant association with participants’ level of knowledge. Women were significantly more knowledgeable and had higher scores (ß: 0.41; 95% CI: 0.13, 0.69) than men (p<0.05). Additionally, participants aged 20-29 years had significantly higher knowledge scores (ß: 0.39; 95% CI: 0.15, 0.63) than younger participants (p<0.05).

Conclusions

Based on our findings, participants’ knowledge and awareness about oral piercing is adequate in general. However, there should be more efforts to educate the people of Riyadh about the complications of these piercings as well as raise awareness about proper oral hygiene methods.

## Introduction

Body piercing was a practice linked with religious or ceremonial rites in antiquity. In modern times, it has gained popularity among teenagers and young people as a form of self-expression or fashion statement. While ear lobes, noses, eyebrows, and navels are common sites for body piercing, the tongue is the most frequently pierced oral location [1].

Many teenagers and young adults get body and oral piercings these days, which can cause several complications [2]. As described in the literature [2], piercings can act as a transfer tool for bacterial diseases such as Neisseria-induced endocarditis, Streptococcus viridans, and Ludwig's angina as well as viruses like HIV, hepatitis A virus, hepatitis B virus, hepatitis C virus, herpes simplex virus, and Epstein-Barr virus [2]. These microorganisms can also lead to oral complications like atypical trigeminal neuralgia, soft tongue tissue lesions, and hypertrophic keloid lesions [2]. Several research studies have documented the negative outcomes, both small and significant, resulting from the piercing of the skin or mucous membranes, or from the sustained presence of oral and perioral piercings [2]. Patients with intraoral piercing often present with poor dental and periodontal health, as well as various complications and side effects, such as discomfort, edema, infection, gingival damage, gingival recession, chipped or cracked teeth, non-carious tooth problems, increased salivation, nickel, chromium, or nickel-cobalt hypersensitivity, as well as difficulty speaking and swallowing [1,3]. The severity of these complications is believed to be related to the piercing design and the duration of having them [3].

The general public, including young people, is often poorly informed about the risks they are exposed to after intraoral piercing and the strategies for minimizing them, and even healthcare professionals often have limited knowledge of risks and complications that may arise after this surgical procedure [4,5,6]. Body piercing must be treated as a surgical procedure, and must only be carried out by competent people capable of ensuring high standards of professionalism in settings subject to sanitary inspections [5]. Additionally, it would be beneficial to offer regular follow-ups to individuals who get body piercings [5]. Although every patient is assumed to have complied with the piercer's instructions, 96% of them reported postoperative local complications, including bleeding within 12 hours of the piercing (90%), perilesional edema for two to three days following piercing surgery (80%), persistent mucosal atrophy (70%), enamel abrasions (30%), enamel fractures (30%), gingival recession (25%), and erythematous palatal mucosa [5]. Every patient who has had their tongue pierced has complained of a temporary change in taste, probably due to the presence of a metallic foreign body in the oral cavity or blood or serum leaks while the tissues are healing [5].

The tongue is the most frequently pierced oral site, and barbell piercings are the most popular type of piercing [6]. Although some piercings are carried out on the dorsolateral side of the tongue anterior to the lingual frenum, the tongue is frequently pierced in the midline, more precisely in the median lingual sulcus [6]. The tongue is highly vascularized and innervated, and controlled bleeding can be expected during piercing [7]. There has been evidence that piercing can cause significant bleeding [7]. Additionally, special care must be taken to avoid damage to any nerves, which may cause paresthesia [7]. Original lesions formed post-piercing on the tongue will disappear after three to five weeks [7]. Local irritation begins six to eight hours after the piercing and peaks on day three or four [7]. Although usually transient, inflammation can last for weeks and can result in granulomatous inflammation as a response to the foreign body, impairing chewing and swallowing and occasionally even causing suffocation or breathing problems [7].

Oral and perioral piercings may cause various diseases as a reaction to the procedure [8]. To ensure early detection of the various negative effects linked to this procedure, people with oral and perioral piercings should see their dentist on a regular basis for a thorough oral checkup [8]. Certain techniques have been demonstrated to minimize the impact of oral piercing [6]. These methods include (a) consuming a cold liquid diet on the first day, followed by a diet of predominantly light, soft food for a few weeks; (b) applying ice to the skin for 30 or 45 minutes five times a day; (c) mouth-washing with 0.12% chlorhexidine after the first day for five times a day for the first 10 days; and (d) reducing alcohol, cigarette, and caffeine intake as these substances hinder epithelial reconstruction [6]. Also, choosing a smaller piece of jewelry for piercing, and paying close attention to oral hygiene, particularly at the piercing site and during the healing period, as well as checking the condition of the oral and perioral piercings as frequently as possible to prevent infections are recommended [6].

Oral piercings come in different shapes; when it comes to the piercing's design, the ball-shaped tip is the most prevalent one (94% of piercings), followed by the cone-shaped tip (4%), with the cylindrical shape being the least popular, accounting for only 2% of piercings [6]. Titanium (65%), steel (25%), acrylic (6.3%), and niobium (5%) are the materials most commonly utilized for piercings [6]. Nickel is also used in piercings, and it is the metal that causes most contact allergies and is found to trigger anaphylactic reactions [7]. Contact dermatitis caused by nickel, chromium, or nickel-cobalt is the most commonly reported allergic reaction post-piercing [7].

In a previous study [8] conducted among school-age students, a high proportion of teenage students reported receiving oral piercing and experiencing some local complications. In another recent study [9], it was found that the periodontal area of teeth near the tongue piercing, such as the ventral surface of the tongue and the lingual surfaces of teeth and implants in the mandibular anterior sextant, is the most jeopardized. Moreover, another study revealed that 17% of people who underwent oral piercings were unable to confirm whether the area that had undergone piercing had been sterilized and disinfected before piercing [1]. From a dental viewpoint, tongue and mouth piercings are not acceptable practices because they frequently result in both local and systemic issues. Depending on when they start, complications may surface quickly or arise slowly [5].

To the best of our knowledge, no published data assessing the knowledge of oral and perioral piercing among the population living in Riyadh City, Saudi Arabia are currently available. Hence, this study aimed to measure the knowledge and awareness of oral and perioral piercing and its complications among the population living in Riyadh City, Saudi Arabia, and evaluate the association of nationality, sex, age, and socioeconomic status (educational level, area of residence, and income) with the level of individual knowledge.

## Materials and methods

This was an observational cross‐sectional study (survey) with a sample size of 1,099 participants. The cross-sectional study design was deemed suitable for the objective of this study. The collection of longitudinal data was not required for this study; therefore, a follow-up questionnaire was not required. The target sample size was estimated using power analyses after consulting with a statistician. A confidence interval (CI) of 95%, a standard deviation of 0.5, and a margin of error of 5% were employed. This study was approved by the Research Ethics Committee of King Khaled University Hospital (KKUH) (Project No: E-22-7177).

The data were collected from participants living in Riyadh, Saudi Arabia. All information regarding the study questionnaire was explained, and informed consent was obtained from each participant. The study was conducted between October 2022 and January 2023. Individuals of both genders who were aged above 13 years and living in Riyadh, Saudi Arabia were included in the study regardless of their nationality. Participants aged less than 13 years old and those not living in Riyadh City were excluded from the study.

A structured questionnaire was used as the study tool, which was developed after consulting relevant studies in the literature [1,2,3,4]. The final version of the questionnaire was validated [1,2,3,4] and reviewed by two reviewers (HA and AT). The questionnaire contained 26 questions and was classified into three main sections: sociodemographic data, awareness and self-perception regarding oral piercing, and knowledge level regarding oral piercing and oral health. The variables analyzed included nationality, sex, age, socioeconomic status (educational level, area of residence, and monthly income), and awareness regarding oral and perioral piercing and its complications.

The questionnaire was distributed in multiple residential areas in Riyadh, such as North, South, Middle, Eastern, and Western Riyadh. The data collection was performed using simple random sampling via electronic questionnaires (Google Forms) distributed to participants living in Riyadh. Social media platforms such as WhatsApp and Twitter as well as emails were used to reach out to this study's desired sample population.

IBM SPSS Statistics version 26 (IBM Corp., Armonk, NY) was used for data analysis. Categorical variables are presented as frequencies and percentages, and continuous variables as means and standard deviations (SD). The oral piercing knowledge score was calculated by summing up the scores of seven questions related to participants' knowledge of oral complications and hygiene practices associated with oral piercing [("Do you think oral piercing can chip any of the teeth? Do you think oral piercing can cause discomfort in the gum? Do you think oral piercing can cause halitosis (bad breath)? Do you think oral piercing can cause inflammation/infection? Do you think once the oral piercing is done, there will be any change in oral hygiene methods? From your point of view, how often should oral jewelry piercing be cleaned? From your point of view, what are the best methods for cleaning an oral piercing?"]. The responses were coded as 0 for "No" and "Do not know", and 1 for "Yes". For responses to questions regarding the frequency of cleaning responses, "cleaning every day" was coded as 1 while other responses were coded as 0. Finally, for responses to the question regarding the best methods for cleansing oral piercing, "cleaning using an antimicrobial solution" was coded as 1, and other responses were coded as 0. The total score ranged from a minimum score of 0 to a maximum score of 7.

## Results

Table 1 shows the sociodemographic characteristics of the study sample. A total of 1,054 individuals completed the survey. Of these, 95.6% were Saudi nationals. Approximately 85.5% of the participants (n = 901) were women, and 52.4% were aged 20-29 years (n = 552). Finally, most participants (62.9%, n = 663) had a low monthly income (< 5,000 SR), and, in terms of residence, the highest number of participants were from the northern region of Riyadh (37.1%, n = 391).

**Table 1:** Sociodemographic characteristics of the study participants (N = 1,054) SAR: Saudi Riyal

Sociodemographic characteristics		N (%)
Nationality	Non-Saudi	46 (4.4)
	Saudi	1,008 (95.6)
Gender	Male	153 (14.5)
	Female	901 (85.5)
Age, years	13-20	256 (24.3)
	20-29	552 (52.4)
	30-39	157 (14.9)
	40-49	49 (4.6)
	50-59	29 (2.8)
	>60	11 (1.0)
Monthly income, SAR	<5000	663 (62.9)
	5,000–10,000	218 (20.7)
	10,000–20,000	102 (9.7)
	20,000–30,000	33 (3.1)
	30,000–40,000	16 (1.5)
	>40,000	22 (2.1)
Region	North Riyadh	391 (37.1)
	South Riyadh	113 (10.7)
	Eastern Riyadh	267 (25.3)
	Western Riyadh	117 (11.1)
	Middle Riyadh	166 (15.7)

Data pertaining to the prevalence of oral piercings and participants' knowledge about it are presented in Table 2. Most participants had heard of or seen an oral or perioral piercing (89.1%, n = 939). However, very few of them had received an oral piercing themselves (10.7%, n = 113) or had a family member with an oral piercing (18.7%, n = 197). Participants reported that the most commonly observed site for oral piercing was the lip (29.8%, n = 314), and teenagers were the most common age group with oral piercings (76.3%, n = 804). Regarding the purpose, style, and component of oral piercing, participants reported that being trendy (32.4%, n = 342) was the most commonly perceived purpose for having an oral piercing; ball captive ring (40.4%, n = 426) was the most common style. Of note, more than two-thirds of the participants (64.8%, n = 683) were not aware of the main component of the jewelry. However, more than two-thirds of participants (62.6%, n = 660) agreed that oral piercing should be performed by personnel with relevant professional qualifications.

**Table 2:** Prevalence of oral piercing and knowledge among study participants (N = 1,054) PMMA: polymethyl methacrylate; PTFE: polytetrafluorethylene; HBV: hepatitis B virus; HSV: herpes simplex virus; HAV: hepatitis A virus; HCV: hepatitis C virus

Knowledge and self-perception of oral piercing		N (%)
Have you ever heard about or seen oral or perioral piercing?	No	115 (10.9%)
	Yes	939 (89.1%)
Have you ever had an oral piercing?	No	941 (89.3%)
	Yes	113 (10.7%)
Did one of your family members have an oral piercing?	No	857 (81.3%)
	Yes	197 (18.7%)
If you have heard about, seen, or had an oral piercing, what type was it?	Lip	314 (29.8%)
	Cheek	106 (10.1%)
	Tongue	155 (14.7%)
	Lip + cheek	53 (5.0%)
	Lip + tongue	144 (13.7%)
	Cheek + tongue	17 (1.6%)
	Lip + cheek + tongue	265 (25.1%)
What do you think is the most common age group that goes for oral piercings?	Children	21 (2.0%)
	Teenagers	804 (76.3%)
	Adults	218 (20.7%
	Elderly	11 (1.0%)
From your point of view, what is the purpose of having an oral piercing?	Esthetic	145 (13.8%)
	Feeling of independence	65 (6.2%)
	Trend	342 (32.4%)
	Other	130 (12.3%)
	Esthetic + feeling of independence	14 (1.3%)
	Esthetic + trend	183 (17.4%)
	Esthetic + others	9 (0.9%)
	Feeling of independence + trend	45 (4.3%)
	Feeling of independence + other	10 (0.9%)
	Trend + other	42 (4.0%)
	Trend + esthetic + feeling of independence	33 (3.1%)
	Esthetic + feeling of independence + trend + other	9 (0.9%)
	Esthetic + trend + other	20 (1.9%)
	Feeling of independence + trend + other	6 (0.6%)
	Other + esthetic + feeling of independence	1 (0.1%)
From your point of view, what style of oral jewelry is the most common?	Don’t know	270 (25.6%)
	Ball captive ring	426 (40.4%)
	Barbell	82 (7.8%)
	Labret	72 (6.8%)
	Ball captive ring + barbell	73 (6.9%)
	Ball captive ring + labret	57 (5.4%)
	Barbell + labret	9 (0.9%)
	Ball captive ring + barbell + labret	65 (6.2%)
From your point of view, what is the main component of oral piercing jewelry?	Don’t know	683 (64.8%)
	Titanium	132 (12.5%)
	Surgical steel	119 (11.3%)
	PMMA, PTFE, bioplast (plastic)	61(5.8%)
	Titanium + surgical steel	31 (2.9%)
	Titanium + PMMA, PTFE, bioplast (plastic)	9 (0.9%)
	Surgical steel + PMMA, PTFE, bioplast (plastic)	11 (1.0%)
	Titanium + surgical steel+ PMMA, PTFE, bioplast (plastic)	8 (0.8%)
If someone asked you about having an oral piercing, would you recommend it?	No	940 (89.2%)
	Yes	114 (10.8%)
Do you think that oral piercing should be done by a person with professional qualifications?	No	161 (15.3%)
	Yes	660 (62.6%)
	Don’t know	233 (22.1%)
Are you aware of the danger of mouth piercing?	No	293 (27.8%)
	Yes	761 (72.2%)
Do you think oral piercing can chip any of the teeth?	No	231 (21.9%)
	Yes	477 (45.3%)
	Don’t know	346 (32.8%)
Do you think oral piercing can cause discomfort in the gum?	No	69 (6.5%)
	Yes	830 (78.7%)
	Don’t know	155 (14.7%)
Do you think oral piercing can cause halitosis (bad breath)?	No	100 (9.5%)
	Yes	764 (72.5%)
	Don’t know	190 (18.0%)
Do you think oral piercing should be removed before visiting a dentist?	No	131 (12.4%)
	Yes	544 (51.6%)
	Don’t know	379 (36.0%)
Do you think your dentist would recognize if you had an oral piercing?	No	48 (4.6%)
	Yes	778 (73.8%)
	Don’t know	228 (21.6%)
Do you think once the oral piercing is done, there will be any change in oral hygiene methods?	No	93 (8.8%)
	Yes	768 (72.9%)
	Don’t know	193 (18.3%)
From your point of view, how often should oral jewelry piercing be cleaned?	Never	268 (25.4%)
	Once a week	128 (12.4%)
	Once a month	27 (2.6%)
	Every day	631(59.9%)
From your point of view, what are the best methods for cleaning an oral piercing?	Don’t know	355 (33.7%)
	Antimicrobial solution	300 (28.5%)
	Brushing	57 (5.4%)
	Both	342 (32.4%)
Do you think oral piercing can cause inflammation or infection?	No	77 (7.3%)
	Yes	806 (76.5%)
	Don’t know	171(16.2%)
Complications of oral piercing might include	Allergic reaction + gingival inflammation	914 (86.7%)
	Gingival inflammation	38 (3.6%)
	Allergic reaction	16 (1.5%)
	Temporary paralysis	13 (1.2%)
	Other	73 (7.0%)
Viral complications of oral piercing might include	Don’t Know	771(73.1%)
	HBV	18 (1.7%)
	HSV	92 (8.7%)
	HAV + HBV + HCV	21 (2.0%)
	Other	152 (14.5%)

Regarding the oral effects of piercing, most participants reported being aware of the danger that oral piercing can cause in the mouth (72.2%, n = 761), the possibility of chipping the teeth (45.3%, n = 477), causing discomfort to the gum (78.7%, n = 830), and causing halitosis (72.5%, n = 764). Regarding piercing and oral hygiene, most participants reported being aware of the change in oral hygiene methods (72.9%, n = 768) and that oral jewelry piercing should be cleaned every day (59.9%, n = 631). Nevertheless, a considerable number of respondents (33.7%, n = 355) did not have sufficient knowledge regarding the best methods for cleaning an oral piercing. Finally, regarding piercing and oral inflammation/infection, more than two-thirds of participants (76.5%, n = 806) reported being aware of complications associated with oral piercing, including inflammation/infection. Allergic reactions and gingival inflammation were the most common complications reported by the participants (86.7%, n = 914). However, more than two-thirds of the participants (73.1%, n = 771) were not aware of the common viral complications of oral piercing.

The crude associations between sociodemographic factors and knowledge score regarding oral/perioral piercings and oral health are presented in Table 3. The analysis employed was crude (unadjusted) analysis. Sex and age were the only factors that showed a significant association with participants’ knowledge scores. Women were significantly associated with higher knowledge scores (ß: 0.41; 95% CI: 0.13, 0.69) than men (p<0.05). Additionally, participants aged 20-29 years had significantly higher knowledge scores (ß: 0.39; 95% CI: 0.15, 0.63) than younger participants (p<0.05). 

**Table 3:** Knowledge score differences based on each variable CI: confidence interval; SAR: Saudi Riyal

Sociodemographic factors	ß (95% CI)	P-value
Gender		
Male	1.00 (Reference)	
Female	0.41 (0.13, 0.69)	0.005
Nationality		
Saudi	1.00 (Reference)	
Non-Saudi	-0.25 (-0.73, 0.24)	0.323
Age, years		
<20	1.00 (Reference)	
20–29	0.39 (0.15, 0.63)	0.002
30–39	-0.20 (-0.53, 0.12)	0.216
40–49	-0.32 (-0.812, 0.18)	0.208
50–59	0.02 (-0.61, 0.64)	0.957
>60	-0.16 (-1.14, 0.82)	0.747
Monthly income, SAR		
<5000	1.00 (Reference)	
5,000–10,000	0.12 (-0.14, 0.37)	0.369
10,000–20,000	0.27 (-0.08, 0.61)	0.126
20,000–30,000	-0.09 (-0.67, 0.48)	0.750
30,000–40,000	-0.38 (-1.20, 0.43)	0.357
>40,000	0.13 (-0.56, 0.83)	0.706
Residence		
North Riyadh	1.00 (Reference)	
South Riyadh	-0.06 (-0.40, 0.28)	0.733
Eastern Riyadh	0.22 (-0.04, 0.47)	0.098
Western Riyadh	-0.12 (-0.46, 0.22)	0.490
Middle Riyadh	0.17 (-0.13, 0.47)	0.272

**Figure 1:**
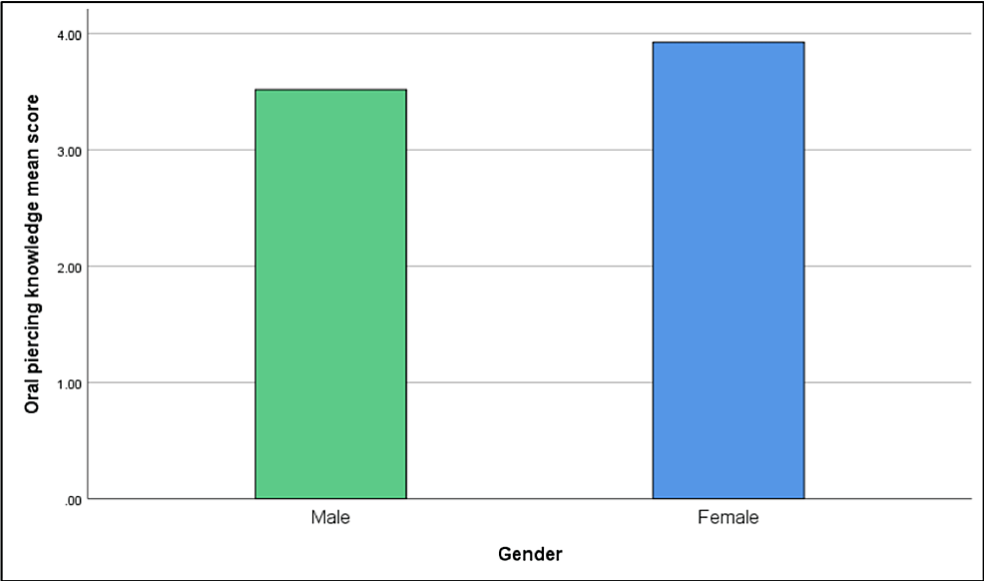
Comparison of Oral piercing knowledge mean score between genders

**Figure 2:**
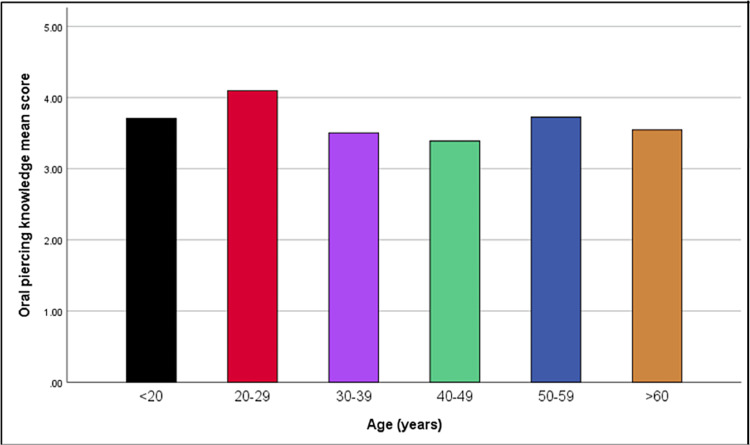
Comparison of oral piercing knowledge mean score among age groups

To summarize, the total mean score for oral piercing knowledge was higher for females (3.93) compared to males (3.52); it was the highest among participants aged 20-29 years (4.09) while participants aged 40-49 years had the lowest mean score (3.39) as shown in Figures 1, 2.

## Discussion

Our study showed that 27.8% of our sample population was not aware of the risks and complications of oral piercings. On the other hand, 86.7% of participants were aware that gingival inflammation and allergic reactions may occur and 45.3% knew that these piercings might cause tooth chipping; 25.4% were not aware that oral jewelry needs to be cleaned regularly. In a study by Reshma et al., 88.2% of the participants had seen or heard of oral piercings, which is consistent with our results, which showed that 89.1% of the sample population were aware of the practice of oral piercings. Regarding the commonly used style of jewelry, Reshma et al.'s results showed that 78.1% were mostly familiar with ball captive rings [3], which is in line with our findings indicating that 40.4% of the study participants were familiar with the same. Also, 74.1% presumed that most oral piercings involved teenagers, which is similar to the results of our study (76.3%). A study conducted in Rome by Vozza et al. [1] showed that there is a lack of knowledge about the risk of cross-infection of viral hepatitis due to oral piercing (74.77%), which is comparable with our study (73.1%). Our study showed that being trendy is usually the main reason for getting an oral piercing (32.4%), which contrasts with a study by Aljameel et al. [4], which stated that esthetics was the main reason (84.1%). However, regarding the most commonly reported site of oral piercing, both studies reported that the lip is the most common site, with Aljameel et al. reporting a figure of 51% while it was 29.8% in our study [4]. Regarding the assumed complications, 78.7% of the participants in our study agreed that gum discomfort might arise, which is in line with the study by Aljameel et al., where 36.5% of participants reported this complication [4]. The majority of the participants (72.5%) in our study presumed that halitosis (bad breath) accompanies oral piercings; however, according to Aljameel et al., only 15.9% reported this complication [4]. 

A study by Inchingolo et al. showed that 60% of teenagers have a piercing [5]. In correlation, our study showed that most participants (76.3%) agreed that teenagers are the age group that most commonly undergo oral piercing. Dentists should also be prepared and well-educated to manage patients with oral piercing, evaluate their oral situation, and prevent any undesirable complications from oral piercings [6]. Escudero-Castaño et al. reported that the most common oral regions that could be affected by oral piercing were the uvula and the side of the tongue, and the most common type of jewelry associated with buccal alterations was the long barbell in the tongue [7]. In correlation, our study showed that 14.7% of the population had seen an oral piercing in the tongue. A study by Firoozmand et al. involving 927 students from private and state schools [8] showed that the tongue was the most common site for oral piercing (66.6%), and complications were observed in 74.3% of the cases [8]. The authors reported that a small percentage of teenage students (3.6%) had an oral piercing, which contrasts with our study results, which showed that teenagers are the most common age group that receives oral piercings (76.3%).

Ibraheem et al. assessed the effect of tongue piercing on periodontal areas and peri-implants and concluded that wearing tongue piercings can increase the risks of peri-implant and periodontal diseases, mainly in the mandibular anterior segment [9]. A review by Hennequin-Hoenderdos aimed to gather knowledge regarding the emergence of gingival recessions and tooth damage. Teeth injuries, including tooth flaws, fractures, chipping, cracks, abrasion, and splitting, are general terms [10]. Tooth injuries are a rather broad description that can include tooth defects, fractures, chipping, cracks, abrasion, and splitting [10]. A tongue piercing increased the incidence of dental damage in individuals by a factor of 2.44. In other words, people with lip piercings are 4.14 times more likely to experience gingival recession than non-pierced individuals.

Piercing procedures are often performed by unlicensed professionals using unsterilized tools. As a result, several cases of piercing failure have been documented globally [3]. According to Hennequin-Hoenderdos et al. [10], the increasing popularity of body piercings among young adults can be attributed to various factors, including the urge to fulfill social demands, establish a personal statement, or enhance sexual appeal; however, Reshma et al. found that 71.3% of students wear oral piercings as a statement of fashion. Also, only 5.8% of students in our study were aware that labrets could be worn as ornaments [3].

Oberholzer et al. found that 59.4% of the participants were unaware of any issues associated with oral piercing [11]. In their study, 17.2% of the respondents had received intraoral piercings five to seven years ago, while 24% had received them within the past year [11]. Most participants reported being aware of the consequences of oral piercings in the mouth. Junco et al.’s study evaluated the effectiveness of educational intervention among dental students and adolescents at schools [12]. Schools provide a favorable environment for enhancing oral health awareness, which could be the first step in modifying negative behavior [12]. Dental students providing information can also be advantageous because teenagers may find themselves in a more laidback atmosphere, which encourages learning and positive response. As health professionals, they may effectively alter the public's understanding and attitudes through education [12]. Adolescents and dental students responded favorably to the educational intervention on mouth piercing. Dental students who actively participated in their education retained significantly more information [12].

In the present study, most participants (72%) were aware of the risk of mouth piercing, 89% did not recommend having oral piercing, and 32% stated that the purpose of having oral piercing was to be trendy. Furthermore, they agreed that piercings should be performed by a qualified piercing practitioner. The present findings are consistent with those of a study by Veeresh et al. [13]. Another study by Ziebolz et al. showed that participants with tongue piercings had poor oral behavior, engaged in insufficient cleaning of piercing, and in most cases, had 80% calculus formation at the piercing surface [14]. Furthermore, allergic reactions and gingival inflammation were the most common complications, which aligns with a previous study by Plessas and Pepelassi [15]. In contrast, in a study by Vieira et al., the most prevalent complications were dental pain and lacerations in the tongue [16]. The trend of getting a piercing as a way of expressing individuality is sometimes stronger than worries about the associated risks; however, we as professionals must respect this need [17]. Professional piercers have an obligation to attend a theoretical and practical training course in anatomy and possible foreseeable complications [17]. Our study validates the data in the literature [6,8,9], which state that intraoral piercings are associated with complications such as allergic reactions, gingival inflammation, infection, chipping of teeth, and halitosis. Moreover, this study supports that oral piercing should be performed by an individual with a relevant professional qualification, which is also supported in the previous literature [3,13].

Our study has a few limitations. Primarily, it was a unicentric study confined to Riyadh city, and hence the findings may not be applicable to other cities in Saudi Arabia. Moreover, we recommend raising awareness among the population, especially teenagers, regarding the complications of oral piercing, as it can significantly improve their oral health as well as encourage them to properly maintain these piercings in the future.

## Conclusions

Our study has demonstrated that the knowledge and awareness about oral piercing among our participants in Riyadh City is adequate as most participants are aware of the practice of oral piercing and its complications. However, more education should be provided regarding the complications of these piercings as well as the proper oral hygiene methods to be followed. Moreover, since teenagers were the most common age group to undergo oral piercing as mentioned by the participants, further awareness and knowledge should be provided to this specific group.

## Appendices

Questionnaire

Section A: Sociodemographic Data

1-What is your gender?

Male 

Female

2-How old are you?

<20 years

20-29 years

30-39 years

40-49 years

50-59 years

>60 years

3-What is your nationality?

Saudi

Non-Saudi

4- How much is your monthly income (in Saudi Riyals)?

5000-10000 SR

10000-20000 SR

20000-30000 SR

30000-40000 SR

>40000 SR

5-Where is your area of residency?

North of Riyadh

South of Riyadh

Eastern Riyadh 

Western Riyadh

Middle Riyadh

*Section B: Awareness and Self-perception Regarding Oral Piercing* 

6-Have you ever heard about or seen oral or perioral piercing? 

Yes

No

7-Have you ever had an oral piercing?

Yes

No

8- Did one of your family members have an oral piercing?

Yes

No

9- If you have heard about, seen, or had an oral piercing, what type was it?

Lip

Cheek 

Tongue

10-What do you think is the most common age group that goes for oral piercings? 

Children

Teenagers

Adults

Elderly

11-From your point of view, what is the purpose of having an oral piercing?

Esthetic

Feeling of independence

Trend

Others…

12-From your point of view, what style of oral jewelry is the most common?

Ball captive ring

Barbell

Labret

Don’t know

13-From your point of view, what is the main component of oral piercing jewelry?

Titanium

Surgical steel

PMMA (polymethyl methacrylate), PTFE (polytetrafluorethylene), bioplast (plastic)

Other…

Don’t know

14-If someone asked you about having an oral piercing, would you recommend it? 

Yes

No

15-Do you think that oral piercing should be done by a person with professional qualifications? 

Yes 

No

Don’t know

16-Do you think oral piercing can chip any of the teeth?

Yes

No

Don’t know

17-Do you think oral piercing can cause discomfort in the gum?

Yes

No

Don’t know

18-Do you think oral piercing can cause halitosis (bad breath)?

Yes

No

Don’t know

19-Do you think oral piercing should be removed before visiting a dentist?

Yes

No

Don’t know

20-Do you think your dentist would recognize if you had an oral piercing?

Yes

No

Don’t know

21-Do you think once oral piercing is done, there will be any change in oral hygiene methods?

Yes 

No

Don’t know

22-From your point of view, how often oral jewelry piercing should be cleaned?

Every day

Once a week 

Once a month 

Never

23-From your point of view, what are the best methods for cleaning an oral piercing?

Antimicrobial solution

Brushing 

Both

24-Do you think oral piercing can cause inflammation or infection?

Yes

No

Don’t know

25-Complications of oral piercing might include. (Multiple answers)

Temporary paralysis

Permanent paralysis

Endocarditis

Ludwig angina

Allergic reaction

Gingival inflammation

Gingival recession

Fractured tooth

Diastema

Hypersalivation

Lingual abscess

26-Viral complications of oral piercing might include. (Multiple answers)

HIV (human immunodeficiency virus)

HAV (hepatitis A virus)

HBV (hepatitis B virus)

HCV (hepatitis C virus)

HSV (herpes simplex virus)
